# Supplementary material for: Empirical methods for controlling false positives and estimating confidence in ChIP-Seq peaks
Source: BMC Bioinformatics. 2008 Dec 5;9:523. doi: 10.1186/1471-2105-9-523 (PMC2628906; doi:10.1186/1471-2105-9-523)
Supplement: Additional File 1 — A variety of html documents from the USeq web site detailing the available applications, their best usage, output file type descriptions, command line menus, etc. [file 1471-2105-9-523-S1.zip › USeqUserGuides/JavaDoc.zip!/JavaDoc/util/bio/annotation/StartStop.html]

StartStop


|  |  |  |
| --- | --- | --- |
| **Overview** \| **Package** \| **Class** \| **Use** \| **Tree** \| **Deprecated** \| **Index** \| **Help** | |  |
| **PREV CLASS**   **NEXT CLASS** | **FRAMES**    **NO FRAMES**     **All Classes** |  |
| SUMMARY: NESTED \| FIELD \| CONSTR \| METHOD | DETAIL: FIELD \| CONSTR \| METHOD |  |


---


## util.bio.annotation Class StartStop

```
java.lang.Object
  util.bio.annotation.StartStop
```

**All Implemented Interfaces:**: java.io.Serializable, java.lang.Comparable

---

``` public class StartStop extends java.lang.Object implements java.lang.Comparable, java.io.Serializable ```

Simple start end object for sorting.

**See Also:**: Serialized Form

---

**Constructor Summary**

`StartStop(int[] startEnd)`

`StartStop(int start,
int end)`


| **Method Summary** | |
| --- | --- |
| `int` | `compareTo(java.lang.Object other)` |
| `int` | `getEnd()` |
| `int` | `getLength()`             Assumes interbase coordinates. |
| `int` | `getStart()` |
| `int[]` | `getStartEnd()` |
| `boolean` | `intersects(int begin, int stop)`             Assumes coordinates are interbase. |
| `static java.util.HashMap<java.lang.String,StartStop[]>` | `parseStartStops(java.io.File bedFile, int subStart, int subEnd)`             Parses a tab delimited file (chr, start, stop, ...). |
| `static int[]` | `startsInBases(StartStop[] ss)`             Returns the starts in number of bases, not coordinates for the array. |
| `java.lang.String` | `toString()` |
| `static int` | `totalBP(StartStop[] ss)`             Returns the total number of bases, assumes interbase coordinates. |

**Methods inherited from class java.lang.Object**

`equals, getClass, hashCode, notify, notifyAll, wait, wait, wait`

**Constructor Detail**

### StartStop

```
public StartStop(int start,
                 int end)
```

---


### StartStop

```
public StartStop(int[] startEnd)
```


**Method Detail**

### toString

```
public java.lang.String toString()
```

:   **Overrides:**: `toString` in class `java.lang.Object`

---


### intersects

```
public boolean intersects(int begin,
                          int stop)
```

:   Assumes coordinates are interbase.

---


### parseStartStops

```
public static java.util.HashMap<java.lang.String,StartStop[]> parseStartStops(java.io.File bedFile,
                                                                              int subStart,
                                                                              int subEnd)
```

:   Parses a tab delimited file (chr, start, stop, ...).

    :   **Parameters:**: `bed` - file, skips empty lines and those starting with '#': `subStart` - and subEnd are the number to subtract from the ends of each region **Returns:**: a HashMap or null in none are found

---


### compareTo

```
public int compareTo(java.lang.Object other)
```

:   **Specified by:**: `compareTo` in interface `java.lang.Comparable`

---


### totalBP

```
public static int totalBP(StartStop[] ss)
```

:   Returns the total number of bases, assumes interbase coordinates.

---


### startsInBases

```
public static int[] startsInBases(StartStop[] ss)
```

:   Returns the starts in number of bases, not coordinates for the array.

---


### getLength

```
public int getLength()
```

:   Assumes interbase coordinates.

---


### getEnd

```
public int getEnd()
```

---


### getStart

```
public int getStart()
```

---


### getStartEnd

```
public int[] getStartEnd()
```


---


|  |  |  |
| --- | --- | --- |
| **Overview** \| **Package** \| **Class** \| **Use** \| **Tree** \| **Deprecated** \| **Index** \| **Help** | |  |
| **PREV CLASS**   **NEXT CLASS** | **FRAMES**    **NO FRAMES**     **All Classes** |  |
| SUMMARY: NESTED \| FIELD \| CONSTR \| METHOD | DETAIL: FIELD \| CONSTR \| METHOD |  |


---
